# Carbonic Anhydrase Inhibitors. Part 54^1^: Metal Complexes of Heterocyclic Sulfonamides: A New Class of Antiglaucoma Agents

**DOI:** 10.1155/MBD.1997.307

**Published:** 1997

**Authors:** Claudiu T. Supuran, Andrea Scozzafava, Andrei Jitianu

**Affiliations:** 1 Università degli Studi Dipartimento di Chimica Laboratorio di Chimica Inorganica e Bioinorganica Via Gino Capponi 7 Florence I-50121 Italy; 2 "I.G. Murgulescu" Institute of Physical Chemistry Academia Romana Spl. Independentei 202 Bucharest R-77208 Romania

## Abstract

Metal complexes of heterocyclic sulfonamides possessing carbonic anhydrase (CA) inhibitory properties were recently shown to be useful as intraocular pressure (IOP) lowering agents in experimental animals, and might be developed as a novel class of antiglaucoma drugs. Here we report the synthesis of a heterocyclic sulfonamide CA inhibitor and of the metal complexes containing main group metal ions, such as Be(II), Mg(II), Al(III), Zn(II), Cd(II) and Hg(II) and the new sulfonamide as well as 5-amino-1,3,4-thiadiazole-2-sulfonamide as ligands. The new complexes were characterized by standard physico-chemical procedures, and assayed as inhibitors of three CA isozymes, CA I, II and IV. Some of them (but not the parent sulfonamides) strongly lowered IOP in rabbits when administered as a 2% solution into the eye.


### CARBONIC ANHYDRASE INHIBITORS. Part 54[1]
### METAL COMPLEXES OF HETEROCYCLIC SULFONAMIDES: A NEW CLASS OF ANTIGLAUCOMA AGENTS

Claudiu T. Supuran[1]*, Andrea Scozzafava[1] and Andrei Jitianu[2]

1 Università degli Studi, Dipartimento di Chimica,
Laboratorio di Chimica Inorganica e Bioinorganica, Via Gino Capponi 7, I-50121, Florence, Italy
2 "I.G. Murgulescu" Institute of Physical Chemistry, Academia Romana,
Spl. Independentei 202, R-77208 Bucharest, Roumania

**Abstract**: Metal complexes of heterocyclic sulfonamides possessing carbonic anhydrase (CA) inhibitory properties were recently shown to be useful as intraocular pressure (IOP) lowering agents in experimental animals, and might be developed as a novel class of antiglaucoma drugs. Here we report the synthesis of a heterocyclic sulfonamide CA inhibitor and of the metal complexes containing main group metal ions, such as Be(II), Mg(II), Al(III), Zn(II), Cd(II) and Hg(II) and the new sulfonamide as well as 5-amino-1,3,4-thiadiazole-2-sulfonamide as ligands. The new complexes were characterized by standard physico-chemical procedures, and assayed as inhibitors of three CA isozymes, CA I, II and IV. Some of them (but not the parent sulfonamides) strongly lowered IOP in rabbits when administered as a 2% solution into the eye.

### Introduction

Sulfonamides possessing carbonic anhydrase (CA, EC 4.2.1.1) inhibitory properties [2] such as acetazolamide **1**, methazolamide **2**, ethoxzolamide **3** and dichlorophenamide **4** have been used for more than 40 years as pressure lowering systemic drugs in the treatment of open-angle glaucoma [3,4]. Their effect is due to inhibition of at least two CA isozymes present within cilliary processes of the eye, ie, CA II and CA IV, which is followed by lowered bicarbonate formation and reduction of aqueous humor secretion [5-7]. Their main drawback is constituted by side effects such as fatigue, augmented diuresis, or paresthesias, due to CA inhibition in other tissues/organs than the target one, ie, the eye [8].

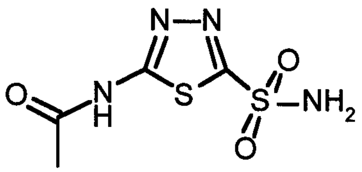

**1**

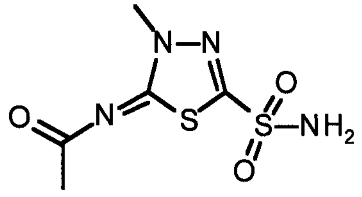

**2**

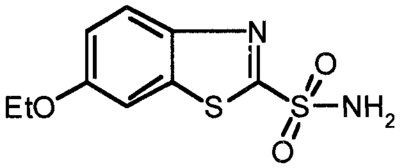

**3**

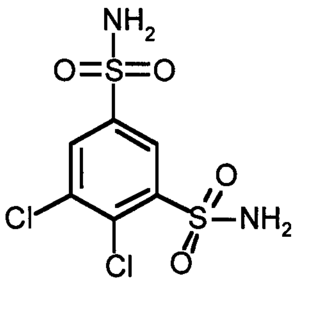

**4**

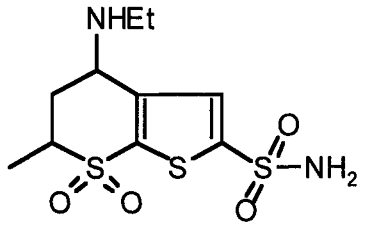

**5**

The above-mentioned side effects are absent in the case in which the inhibitor has topical activity, and is applied directly into the eye. This route has been demonstrated only in 1983 by Maren's group [9] and was followed by the development of the first clinical agent of this type, dorzolamide **5** [10,11]. Dorzolamide (Trusopt) has been introduced in clinical use in 1995 in USA and Europe and it constituted the beginning of a radically new treatment of glaucoma, devoid of the severe side effects observed with the systemic inhibitors [4-6]. The success of topical antiglaucoma CA inhibitors fostered much research in the synthesis and clinical evaluation of other types of such compounds [12-15].

On the other hand, metal complexes of heterocyclic sulfonamides of type **1-5** have been recently prepared by two groups [16-20], and it was proved that they possess much stronger CA inhibitory properties than the sulfonamides from which they were prepared [18-22]. Although the mechanism of CA inhibition of the metal complexes is presently unknown, it was hypothesized that their increased inhibitory power might be due to two processes, occurring separately or in concert, ie, (i) dissociation of the complex inhibitor in sulfonamide anions and metal ions (in diluted solution), which in turn both interact thereafter with the enzyme, at different binding sites, and (ii) direct interaction of the undissociated complex with the enzyme, and more specifically with the hydrophilic patch at the entrance of CA II active site [23], this being the isozyme most susceptible to inhibition with this class of compounds [2,22]. Whether initially the first mechanism of action mentioned above was favored by us [22], recent evidences suggested that the undissociated complex might be the inhibitory species, at least for some isozymes [24]. Since metal complexes are much more inhibitory than the parent sulfonamide from which they were prepared, it appeared of interest to test whether this property might be useful for their use in lowering IOP in experimental animals and whence as a possible glaucoma therapy. Recently we proved [25, 26] that some metal complexes of heterocyclic sulfonamides (which themselves do not possess IOP lowering properties) act as very powerful such agents when administered as diluted solutions directly into the eye of experimental animals, and would thus offer the possibility of developing such totally novel drugs.

Here we report the synthesis of a heterocyclic sulfonamide possessing strong CA inhibitory properties, ie, 5-(chloroacetamido)-1,3,4-thiadiazole-2-sulfonamide, and of the metal complexes of this sulfonamide and of 5-amino-1,3,4-thiadiazole-2-sulfonamide, containing some main group metal ions. The new compounds have been characterized by standard physico-chemical procedures, and were assayed as inhibitors of three CA isozymes, hCA I, hCA II and bCA IV (h = human; b = bovine; these are the isozymes considered to play a critical role in aqueous humour secretion within the eye of higher vertebrates [2-5]).

### Materials and Methods

Melting points were recorded with a heating plate microscope and are not corrected. IR spectra were recorded in KBr pellets with a Carl Zeiss IR-80 instrument. $^1$H-NMR spectra were recorded in DMSO-$d_6$ as solvent, with a Bruker CPX200 instrument. Chemical shifts are reported as values, relative to $Me_4Si$ as internal standard. Conductimetric measurements were done at room temperature (1 mM concentration of complex) in DMSO solution with a Fisher conductimeter. Elemental analyses were done by combustion for C, H, N with an automated Carlo Erba analyzer, and gravimetrically for the metal ions, and were 0.4% of the theoretical values. Thermogravimetric measurements were done in air, at a heating rate of 10°C/min., with a Perkin Elmer 3600 thermobalance.

Sulfonamides used as standards in the enzymatic assay (except for **5**), acetazolamide, pyridine, and chloroacetyl chloride used for the preparation of compound 7, solvents as well as inorganic reagents were from Sigma, Merck and Carlo Erba. 5-Amino-1,3,4-thiadiazole-2-sulfonamide **6** was prepared from acetazolamide by literature procedures [27], by desacetylation with concentrated hydrochloric acid, followed by neutralization with sodium bicarbonate of the corresponding hydrochloride (Scheme 1). Dorzolamide hydrochloride **5** was from Merck, Sharp and Dohme or was prepared as described by Ponticello et al [10,11].

Human CA I and CA II cDNAs were expressed in *Escherichia coli* strain BL21 (DE3) from the plasmids pACA/hCA I and pACA/hCA II described by Forsman et al. [28] (the two plasmids were a gift from Prof. Sven Lindskog, Umea University, Sweden). Cell growth conditions were those described by Lindskog's group [29], and enzymes were purified by affinity chromatography according to the method of Khalifah et al [30]. Enzyme concentrations were determined spectrophotometrically at 280 nm, utilizing a molar absorptivity of 49 $mM^{-1}.cm^{-1}$ for hCA I and 54 $mM^{-1}.cm^{-1}$ for hCA II, respectively, based on $M_r$ = 28.85 kDa for hCA I, and 29.3 kDa for hCA II, respectively [31,32]. bCA IV was isolated from bovine lung microsomes as described by Maren et al, and its concentration has been determined by titration with ethoxzolamide [33].

*Synthesis of 5-(chloroacetamido)-1,3,4-thiadiazole-2-sulfonamide* 7

An amount of 1.80 g (10 mmol) of 5-amino-1,3,4-thiadiazole-2-sulfonamide **6** was suspended in 20 mL of anhydrous acetonitrile and 0.9 mL (0.87g, 11 mmol) of pyridine added. The mixture was magnetically stirred at 4 °C for 10 minutes, then 10.5 mmol of monochloroacetyl chloride, dissolved in 3 mL acetonitrile, were added dropwise for 5 min, and stirring was continued for other 2 hours at room temperature. After an additional 30 min of refluxation, followed by cooling, the precipitated crystals were filtered and recrystallized from ethanol. Yield of 62% white crystals, mp 246-248 ° lit [34] mp ; IR (KBr), $cm^{-1}$: 590, 610, 660, 790, 935, 1090, 1115, 1170, 1350, 1400, 1550, 1650, 1720, 2870, 3280 - 3370 (broad); UV spectrum, $\lambda_{max}$, nm (lg): 255 (3.50); 288 (4.37) $^1$H-NMR (DMSO-$d_6$), δ, ppm: 2.96 (s, 2H, $CH_2$); 8.20 (s, 2H, $SO_2NH_2$); 12.22 (s, 1H, CONH). Analysis, found: C, 18.56; H, 1.88; N, 21.76; S, 24.62 %; $C_4H_5ClN_4O_3S_2$ requires: C, 18.72; H, 1.96; N, 21.83; S, 24.98 %.

*General procedure for the preparation of compounds* **8-20**

An amount of 6 mmol of sodium salt of sulfonamides **6** or **7** was prepared by reacting the corresponding sulfonamide with the required amount of an alcoholic 1N NaOH solution, in ethanol as solvent. To this solution was added the aqueous metal salt (Zn(II), Mg(II), Al(III), Cd(II) chlorides, and Be(II), Pb(II) and Hg(II) nitrate) solution, working in molar ratios $RSO_2NH^-$ : $M^{n+}$ of 2:1 for the divalent cations and 3:1 for the trivalent cation, respectively. The aqueous-alcoholic reaction mixture was heated on a steam bath for one hour, adjusting the pH at 7 if necessary, and after being cooled at 0 °C the precipitated complexes were filtered and thoroughly washed with alcohol-water 1:1 (v/v) and air dried. Yields were in the range of 85-90 %. The obtained white powders of compounds **8-20** melted with decomposition at temperatures higher than 350 °C, and were poorly soluble in water and alcohol, but had good solubilities in DMSO, DMF as well as mixtures of DMSO-water, DMF-water.

***Pharmacology***

*Carbonic anhydrase inhibition*

Initial rates of 4-nitrophenyl acetate hydrolysis catalysed by different CA isozymes were monitored spectrophotometrically, at 400 nm, with a Cary 3 instrument interfaced with an IBM compatible PC [35]. Solutions of substrate were prepared in anhydrous acetonitrile; the substrate concentrations varied between $2.10^{-2}$ and $1.10^{-6}$ M, working at 25°C. A molar absorption coefficient of 18,400 $M^{-1}.cm^{-1}$ was used for the 4-nitrophenolate formed by hydrolysis, in the conditions of the experiments (pH 7.40), as reported in the literature [35]. Non-enzymatic hydrolysis rates were always subtracted from the observed rates. Duplicate experiments were done for each inhibitor concentration, and the values reported throughout the paper are the mean of such results. Stock solutions of inhibitor (1 mM) were prepared in distilled-deionized water with 10-20% (v/v) DMSO (which is not inhibitory at these concentrations [2]) and dilutions up to 0.01 nM were done thereafter with distilled-deionized water. Inhibitor and enzyme solutions were preincubated together for 10 min at room temperature prior to assay, in order to allow for the formation of the E-I complex. The inhibition constant $K_I$ was determined as described by Pocker and Stone [35]. Enzyme concentrations were 3.3 nM for hCA II, 10 nM for hCA I and 34 nM for bCA IV (this isozyme has a decreased esterase activity [36] and higher concentrations had to be used for the measurements).

*Measurement of tonometric IOP*

Adult male New Zealand albino rabbits weighing 2-3 kg were used in the experiments (three animals were used for each inhibitor studied). The experimental procedures conform to the Association for Research in Vision and Ophthalmology Resolution on the use of animals. The rabbits were kept in individual cages with food and water provided *ad libitum*. The animals were maintained on a 12 h: 12 h light/dark cycle in a temperature controlled room, at 22-26 °C. Solutions of inhibitors (2 %, by weight) were obtained in DMSO-water (2:3, v/v) due to the low water solubility of some of these derivatives. Control experiments with DMSO (at the same concentration as that used for obtaining the inhibitors solutions showed that it does not possess IOP lowering or increasing effects.

IOP was measured using a Digilab 30R pneumatonometer (BioRad, Cambridge, MA, USA) as described by Maren's group [37-39]. The pressure readings were matched with two-point standard pressure measurements at least twice each day using a Digilab Calibration verifier. All IOP measurements were done by the same investigator with the same tonometer. One drop of 0.2 % oxybuprocaine hydrochloride (novesine, Sandoz) diluted 1:1 with saline was instilled in each eye immediately before each set of pressure measurements. IOP was measured three times at each time interval, and the means reported. IOP was measured first immediately before drug administration, then at 30 min after the instillation of the pharmacological agent, and then each 30 minutes for a period of several hours. For all IOP experiments drug was administered to only one eye, leaving the contralateral eye as an untreated control. The ocular hypotensive activity is expressed as the average difference in IOP between the treated and control eye, in this way minimizing the diurnal, seasonal and interindividual variations commonly observed in the rabbit [37-39]. All data are expressed as mean SE, using a one-tailed *t* test.

**Results and Discussion**

Reaction of 5-amino-1,3,4-thiadiazole-2-sulfonamide **6** [19b] with chloroacteyl chloride in the presence of pyridine afforded 5-(chloroacetamido)-1,3,4-thiadiazole-2-sulfonamide **7**, by the procedure already reported by Young et al. [34] (Scheme 1).

The sulfonamide **7** has been characterized by elemental analysis and spectroscopic methods which confirmed its structure (only its m.p. has been reported in ref. [34]). The sodium salt of sulfonamides **6** and **7**, obtained *in situ* from the corresponding sulfonamide and sodium hydroxide, were then used for the preparation of coordination compounds, containing the following metal ions: Be(II), Mg(II), Al(III), Zn(II), Cd(II) and Hg(II). Mention should be made that although 5-amino-1,3,4-thiadiazole-2-sulfonamide **6** is the parent compound of important sulfonamide CA inhibitors, such as acetazolamide, benzolamide, methazolamide, etc., its coordination chemistry has been scarcely investigated up to now [22, 40].

The new complexes prepared in this work are shown in Table I. Both compounds containing the sulfonamide-deprotonated species of sulfonamide **7** (LH), as well as complexes in which the anion of 5-amino-1,3,4-thiadiazole-2-sulfonamide (tda) act as ligands, have been prepared. In fact in another work [40] it was documented that in some cases, sulfonamides derived from this ring system may undergo hydrolysis to the moiety substituting the 5 position, with the formation of 5-amino-1,3,4-thiadiazole-2-sulfonamide **6**, which thereafter coordinates metal ions present in solution.

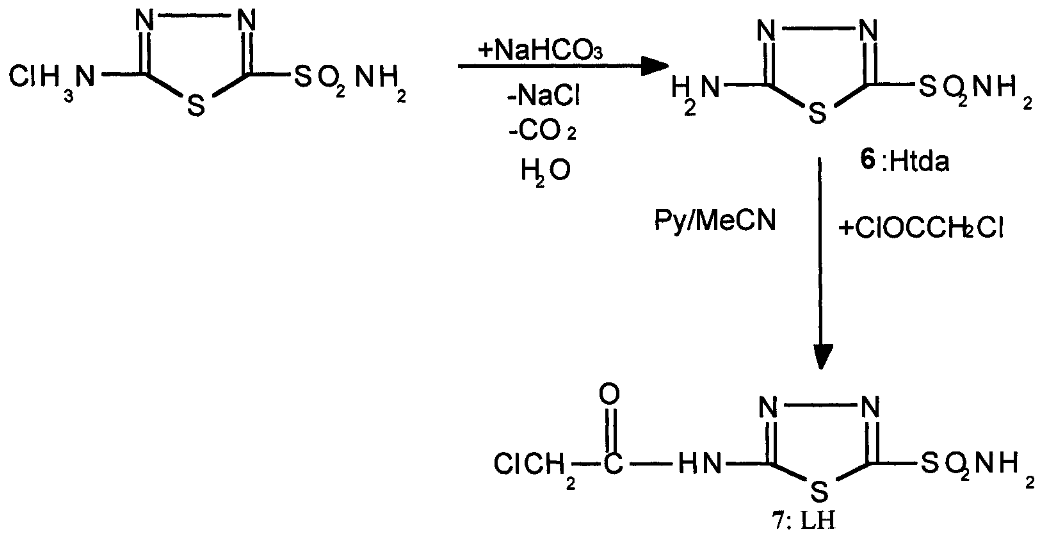

**Scheme 1**

Thus, the X-ray crystal structure of the complex $[Zn(tda)_2(NH_3)].H_2O$ prepared in this way has recently been reported by this group [40]. On the other hand, when the ligand **7** has not been hydrolyzed (during the preparation of the coordination compounds) in the presence of the metal ion to 5-amino-1,3,4-thiadiazole-2-sulfonamide and chloroacetate, the metal complexes contining **6** as ligand have been prepared from the last (pure) compound (as sodium salt) and the corresponding metal salt, by the general procedure described in the Experimental part.

Table I: Prepared complexes **8-20**, containing the conjugate bases of sulfonamides **6** and **7** as ligands and their elemental analysis data. L stands for the sulfonamide deprotonated species of 7, whereas tda for the sulfonamide deprotonated species of 5-amino-1,3,4-thiadiazole-2-sulfonamide **6**.

| No. | Complex | Yield (%) | Analysis (calculated/found) %M[a] | %C[b] | %H[b] | %N[b] |
|---|---|---|---|---|---|---|
| **8** | $[Be(tda)_2]$ | 78 | 2.4/2.5 | 13.0/13.1 | 1.6/1.3 | 30.4/30.2 |
| **9** | $[Mg(tda)_2].3\ H_2O$ | 76 | 5.5/5.1 | 11.0/10.8 | 2.7/2.3 | 25.6/25.5 |
| **10** | $[Zn(tda)_2]$ | 83 | 15.4/15.0 | 11.3/11.4 | 1.4/1.1 | 26.4/26.4 |
| **11** | $[Cd(tda)_2]$ | 90 | 23.8/24.1 | 10.2/10.1 | 1.2/1.2 | 13.7/23.3 |
| **12** | $[Hg(tda)_2]$ | 95 | 35.8/35.7 | 8.5/8.1 | 1.0/1.2 | 20.0/19.8 |
| **13** | $[Pb(tda)_2(OH_2)_2]$ | 84 | 34.4/34.7 | 7.9/7.9 | 1.6/1.3 | 18.6/18.5 |
| **14** | $[Al(tda)_3]$ | 72 | 4.7/4.4 | 12.7/12.8 | 1.6/1.6 | 29.7/29.6 |
| **15** | $[BeL_2]$ | 75 | 1.7/1.6 | 18.4/18.1 | 1.5/1.5 | 21.5/21.3 |
| **16** | $[AlL_3]$ | 59 | 3.4/3.5 | 18.1/17.9 | 1.5/1.3 | 21.1/20.8 |
| **17** | $[ZnL_2]$ | 87 | 11.3/11.5 | 16.6/16.2 | 1.3/1.4 | 19.4/19.3 |
| **18** | $[CdL_2]$ | 88 | 18.0/18.1 | 15.3/14.9 | 1.2/1.1 | 17.9/17.8 |
| **19** | $[HgL_2]$ | 92 | 28.1/28.3 | 13.4/13.3 | 1.1/1.2 | 15.7/15.6 |
| **20** | $[PbL_2(OH_2)_2]$ | 95 | 27.4/27.2 | 12.7/12.5 | 1.0/1.0 | 14.8/14.6 |

[a]By gravimetry; [b]By combustion.

The new complexes have also been characterized by spectroscopic, conductimetric and thermogravimetric measurements (Table II). By comparing the IR spectra of the complexes and the corresponding ligands, the following observations should be made: (i) the shift of the two sulfonamido vibrations (both the symmetric as well as the the assymetric one), towards lower wavenumbers in the spectra of the complexes, as compared to the spectra of the corresponding ligand (Table II), as already documented previously for similar complexes [13-22]. This is a direct indication that the deprotonated sulfonamido moieties of the ligands interacts with the metal ions in the newly prepared coordination compounds; (ii) the amide vibrations (the most intense such bands at 1670-1680 $cm^{-1}$) of ligand **7** appear unchanged in the IR spectra of complexes **15-20** (data not shown), suggesting that these moieties do not participate in coordination of the metal ions; (iii) the C=N stretching vibration in the spectra of the prepared complexes is shifted with 5-20 $cm^{-1}$ towards lower wavenumbers, as compared to the same vibration in the spectra of sulfonamides **6** and **7**, indicating that one of the endocyclic nitrogens of the thiadiazolic ring (presumably N-3) acts as donor atom, as already documented by X-ray crystallographic and spectroscopic determinations on complexes of other sulfonamides (such as **1-3**) with divalent metal ions [13-22] (Table II); (iv) changes in the region 3100-3160 $cm^{-1}$, as the bands present in the spectra of sulfonamides **6, 7** are present in the spectra of complexes **8-20** too, but they are not well resolved, and have a smaller intensity. This is probably due to deprotonation of the $SO_2NH_2$ moiety and participation in the binding of cations; (v) the amino vibrations from 3320 $cm^{-1}$ in the spectra of **6** appear unchanged in the spectra of its complexes **8-14** (data not shown).

In the $^1$H-NMR spectra of compound **6** and its metal complexes, the signal of the amino group has been evidenced as a broad singlet centered at 4.54 ppm (Table II), which is not exchangeable by addition of $D_2O$ into the NMR tube, in contrast to the sulfonamido $NH_2$ protons (which readily exchange). This proves that the 5-amino moiety is not involved in binding the metal ions, as already shown in the X-ray crystallographic work of the complex $[Zn(tda)_2(NH_3)].H_2O$ previously reported [40]. For sulfonamide **7** the CONH proton resonates as a singlet at 12.22 ppm. In complexes **15-20** only very minor shifts of this signal were evidenced (Table II), proving basically that the CONH moiety does not interact with the metal ions in these complexes.

Table II: Spectroscopic, thermogravimetric and conductimetric data for compounds **6-20**.

| Comp. | IR Spectra[a], $cm^{-1}$ $\nu(SO_2)^s$; $\nu(SO_2)^{as}$ | $\nu$(C=N) | $^1$H-NMR Spectra[b] CONH, $\delta$ (ppm) | TG analysis[c] calc./found | Conductimetry[d] $\Lambda_M$ ($\Omega^{-1}$ x $cm^2$x $mol^{-1}$) |
|---|---|---|---|---|---|
| **6** | 1170; 1350 | 1610 | A | e | 2 |
| **8** | 1150; 1300 | 1600 | A | e | 7 |
| **9** | 1150; 1305 | 1600 | A | 12.3/12.1[f] | 4 |
| **10** | 1145; 1300 | 1600 | A | e | 5 |
| **11** | 1145; 1305 | 1600 | A | e | 3 |
| **12** | 1140; 1300 | 1590 | A | e | 2 |
| **13** | 1145; 1300 | 1605 | A | 5.9/5.7[g] | 2 |
| **14** | 1150; 1300 | 1605 | A | e | 6 |
| **7** | 1170; 1350 | 1610 | 12.22 (1H) | e | 3 |
| **15** | 1130; 1335 | 1605 | 12.19 (2H) | e | 4 |
| **16** | 1140; 1330 | 1610 | 12.18 (3H) | e | 2 |
| **17** | 1140; 1330 | 1610 | 12.20 (2H) | e | 9 |
| **18** | 1150; 1330 | 1605 | 12.18 (2H) | e | 3 |
| **19** | 1140; 1335 | 1600 | 12.19 (2H) | e | 2 |
| **20** | 1145; 1330 | 1600 | 12.21 (2H) | 4.7/4.8[g] | 8 |

[a] In KBr; [b]In DMSO-$d_6$; A - the signal of the 5-amino group of **6** (appearing in the ligand at 4.54 ppm as a broad singlet) appears at the same chemical shift (4.50 - 4.55 ppm) in complexes **8-14**; [c]Weight loss between 70-250 °C; [d] 1 mM solution, in DMF, at 25°C; [e] No weight loss seen under 250 °C; [f]Corresponding to three lattice water molecules lost at 70-110°C, and [g]Corresponding to two coordinated water molecules, lost at 160-180 °C.

Thermogravimetric analysis showed the presence of uncoordinated water molecules in the molecule of complex **9** (the three waters were lost in a single step, between 70-110 °C) and of coordinated water in the molecules of the lead(II) derivatives **13** and **20**. All these compounds behaved as non-electrolytes in DMF as solvent (Table II). Mention should be made that the Mg(II) complex of sulfonamide **7** could not be isolated. Instead, only the correponding complex of 5-amino-1,3,4-thiadiazole has been obtained from reaction mixtures containing magnesium salts and the sodium salt of **7**, probably due to a metal ion assisted hydrolysis of **7** to **6** and chloroacetate. Generally such hydrolytic processes involve highly acidic conditions and prolonged heating of the 5-alkylamido-1,3,4-thiadiazole-2-sulfonamide derivatives [41], but they might become milder by taking into account the putative catalytic effect of $Mg^{2+}$ ions reported here.

The data shown above lead to the conclusion that ligand **7** shares a common coordination chemistry with acetazolamide **1** with which it is structurally related, whereas **6** probably also behaves similarly to acetazolamide in the sense that the 5-amino group seems not to be involved in coordinating metal ions, at least in the complexes reported by us here (and also in the compound characterized by X-ray crystallography mentioned above [40]). Thus, in all complexes reported here these sulfonamides (as monodeprotonated species at the $SO_2NH_2$ moieties) act as bidentate ligands, through the endocyclic N-3 and the $NH^-$ groups. The proposed formulae of the new complxes are shown below. Except for the two Pb(II) complexes **13** and **20**, as well as the Al(III) derivatives **14** and **16**, which presumably are pseudo-octahedral, the other derivatives are supposed to contain tetrahedral M(II) ions.

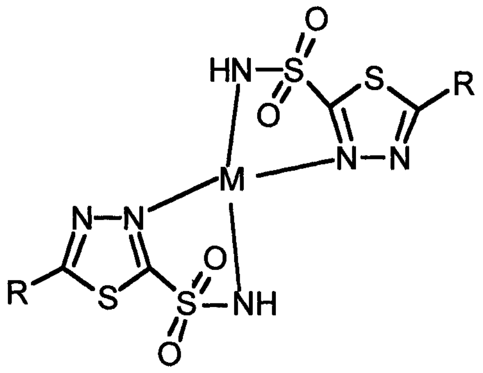

**8-12, 15, 17-19**
R = $H_2N$
R = $ClCH_2CONH$

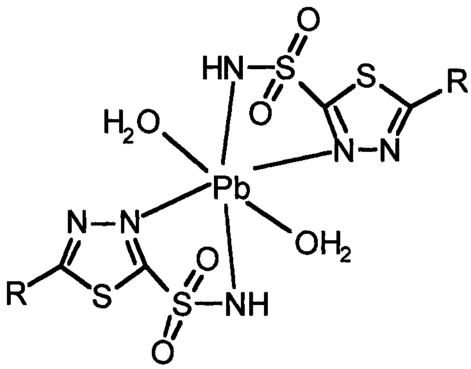

**13**: R = $H_2N$

**20**: R = $ClCH_2CONH$

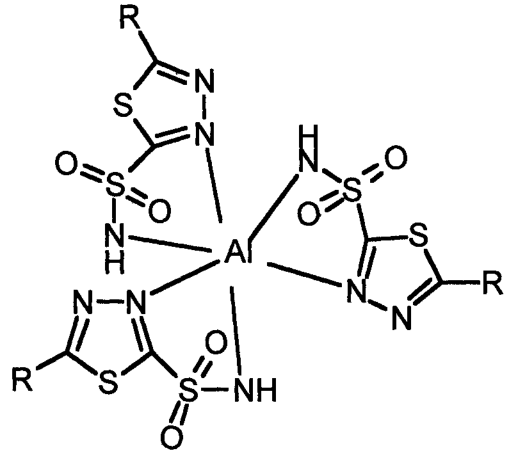

**14**: R = $H_2N$

**16**: R = $ClCH_2CONH$

The compounds **6-20** together with the standard CA inhibitors **1-5** were assayed for inhibition against three isozymes, hCA I, hCA II and bCA IV (Table III). As seen from the above data, the chloracetamido derivative is more inhibitory than acetazolamide, methazolamide and dichlorophenamide, whereas the unacylated compound **6** is less inhibitory than the above sulfonamides. The metal complexes **8-20** are much more inhibitory than the sulfonamides from which they derive **6**, **7** and than all other simple sulfonamides assayed. They behave similarly to the metal complexes of acetazolamide, methazolamide or dorzolamide previously reported by this group, which were all more inhibitory than the parent sulfonamide from which were prepared [16-22, 40]. Particularly strong inhibition was observed for the Zn(II), Hg(II), Pb(II) and Cd(II) complexes, especially against CA II and CA IV, the isozymes critical for aqueous humor formation.

*In vivo* IOP lowering experiments were done in rabbits with some of the new compounds prepared in the present work, such as the sulfonamides **6** and **7**, and their Zn(II) complexes, which were among the strong CA II and CA IV inhibitors in the obtained series. Some of the IOP lowering data at half an hour and one hour after the instillation of one drop of 2 % solution of inhibitor within the rabbit eye are shown in

Table IV, with dorzolamide (at the same concentration) as standard. In Fig. 1 the time dependence of IOP lowering with dorzolamide **5** and the two Zn(II) complexes **10** and **17** is presented.

Table III. CA inhibition data with the standard inhibitors **1-5**, the sulfonamides **6** and **7**, and their metal complexes **8-20**.

| No | Inhibitor | | $K_I$ (nM) | |
|---|---|---|---|---|
| | | hCA I[a] | hCA II[a] | bCA IV[b] |
| **1** | Acetazolamide | 900 | 12 | 220 |
| **2** | Methazolamide | 780 | 14 | 240 |
| **3** | Ethoxzolamide | 25 | 8 | 13 |
| **4** | Dichlorophenamide | 1200 | 38 | 380 |
| **5** | Dorzolamide | >50,000 | 9 | 43 |
| **6** | | 1550 | 230 | 780 |
| **7** | | 640 | 5 | 24 |
| **8** | | 1050 | 190 | 540 |
| **9** | | 350 | 110 | 220 |
| **10** | | 50 | 15 | 25 |
| **11** | | 40 | 14 | 19 |
| **12** | | 12 | 7 | 10 |
| **13** | | 80 | 10 | 26 |
| **14** | | 240 | 76 | 110 |
| **15** | | 120 | 5 | 12 |
| **16** | | 80 | 4 | 16 |
| **17** | | 40 | 3 | 9 |
| **18** | | 40 | 3 | 10 |
| **19** | | 9 | 2 | 5 |
| **20** | | 15 | 5 | 10 |

[a] Human (cloned) isozymes; [b] From bovine lung microsomes.

Table IV: IOP lowering following topical application of CA inhibitors, half an hour and one hour after instillation into the eye of a drop (50 L) of 2 % solution of inhibitor.

| Inhibitor | IOP SE [a] (mm Hg) | |
|---|---|---|
| | 1/2 h | 1h |
| Dorzolamide **5** | 2.2 0.10 | 4.1 0.15 |
| **6** | 0 0.10 | 0 0.09 |
| **7** | 0 0.10 | 0 0.09 |
| **10** | 2.00.09 | 5.0 0.12 |
| **17** | 8.0 0.14 | 8.1 0.21 |

[a] $IOP = IOP_{control\ eye} - IOP_{treated\ eye}$ (N = 3).

As seen from the above data, the sulfonamides 6 and 7 are totally ineffective as IOP lowering agents, similarly to the classical clinically used inhibitors of type 1-5 [2,3]. On the other hand, dorzolamide, the first topical sulfonamide used clinically in the treatment of glaucoma is an effective such agent, with a decrease of IOP of around 4 mm Hg, one hour after administration directly into the eye (Table IV). From the data of this table, it is obvious that the metal complexes of heterocyclic sulfonamides investigated by us behave as much more effective IOP lowering agents than dorzolamide. and their effect is generally longer-lasting (Fig. 1).

A last remark should be made about the possible mechanism of action of the new class of IOP lowering agents. Obviously, their activity is due to inhibition of CA isozymes present in the cilliary processes within the eye, similarly to other topically active sulfonamides [2-6]. The fact that the sulfonamide per se is *inactive* via the topical route, whereas the metal complexes result much better than the drug dorzolamide, indicates that the presence of metal ions in the molecules of these CA inhibitors is essential and confers them completely new properties.

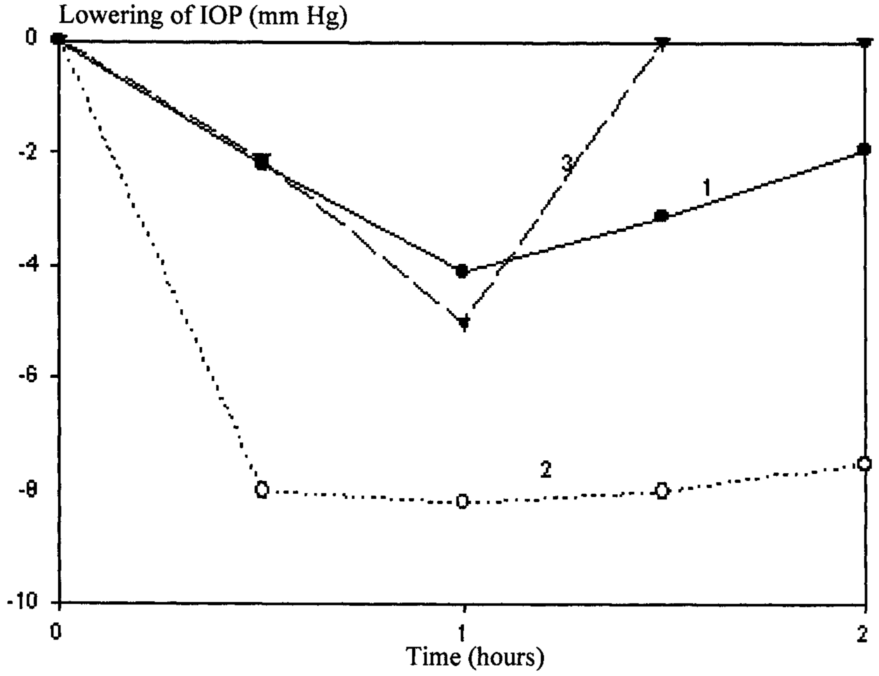

Fig 1: Time dependence of IOP lowering with dorzolamide (curve 1); the zinc complex **10** (curve 3) and the zinc complex **17** (curve 3), after topical administration of one drop of 2 % solution of inhibitor in rabbit.

Preliminary results from this laboratory indicate that the metal complexes of topically active sulfonamides show also increased IOP lowering effects with respect to the complexes prepared in the present study [42]. Our hypothesis is that the presence of the metal ion in the molecules of these complex inhibitors induces a dramatic change in their physico-chemical properties as compared to those of the parent sulfonamide. This phenomenon is certainly governed by the strong polarization induced by the metal ions. In this way, it is quite probable that the right balance between the lipo- and hydrosolubility of these compounds is achieved, which has been considered to be the critical factor for not observing topical activity in the classical CA inhibitors, such as acetazolamide, methazolamide and ethoxzolamide, which were either too lipophilic or too hydrosoluble [2,3]. So, by choosing different metal ions and diverse sulfonamides, much larger possibilities arise to finely tune the pharmacological properties which strongly influence the value of a drug.

In conclusion we describe here a novel class of IOP lowering agents, ie, the metal complexes of sulfonamide CA inhibitors. These derivatives appear to be very active and longer lasting than the drug dorzolamide, and might constitute the premises for a new generation of antiglaucoma drugs.

**Acknowledgments.** We are grateful to Prof. S. Lindskog (Umea Univ., Sweden) for the gift of the hCA I and II plasmids.

**References**

1 Preceding part of this series: Supuran CT, Scozzafava A, Ilies MA, Iorga B, Cristea T, Chiraleu F, Banciu MD (1997) *Eur J Med Chem*, submitted.
2 Supuran CT (1994) "Carbonic anhydrase inhibitors" In *Carbonic Anhydrase and Modulation of Physiologic and Pathologic Processes in the Organism*, (Puscas I, Ed) Helicon, Timisoara, pp. 29-111.
3 Maren TH (1991) "The links among biochemistry, physiology and pharmacology in carbonic anhydrase mediated systems". In *Carbonic Anhydrase - From Biochemistry and Genetics to Physiology and Clinical Medicine*, (Botré F, Gros G, Storey BT Eds) VCH, Weinheim, pp. 186-207.
4 Bayer A, Ferrari F, Maren TH, Erb C (1996) *J Fr Ophtalmol* **19**, 357-362.
5 Maren TH, Conroy CW, Wynns GC, Levy NS (1997) *J Ocul Pharmacol Therapeut* **13**, 23-30.
6 Sugrue MF (1996) *J Ocul Pharmacol Ther* **12**, 363-376.
7 Bartlett JD, Jaanus SD (1989) "Carbonic anhydrase inhibitors". In *Clinical Ocular Pharmacology*, Second Edition, Butterworths Publishers, Boston, pp. 254-263.
8 Alward PD, Wilensky JT (1981) *Arch Ophthalmol* **99**, 1973-1976.

9 Maren TH, Jankowska L, Sanyal G, Edelhauser HF (1983) *Exp Eye Res* **36**, 457-480.
10 a) Ponticello GS, Freedman MB, Habecker CN, Lyle PA, Schwam H, Varga SL, Christy ME, Randall WC, Baldwin JJ(1987) *J Med Chem* **30**, 591-597; b) Greer J, Erickson JW, Baldwin JJ, Varney MD (1994) *J Med Chem* **37**, 1035-1054.
11 Baldwin JJ, Ponticello GS, Anderson GS, Christy ME, Murcko MA, Randall WC, Schwam H, Sugrue MF, Springer JP, Gautheron P, Grove J, Mallorga P, Viader MP, McKeever BM, Navia MA (1989) *J Med Chem* **32**, 2510-2513.
12 Chow K, Lai R, Holmes JM, Wijono M, Wheeler LA, Garst ME (1996) *Eur J Med Chem* **31**, 175-186.
13 Supuran CT, Nicolae A, Popescu A (1996) *Eur J Med Chem* **31**, 431-438.
14 Supuran CT, Popescu A, Ilisiu M, Costandache A, Banciu MD (1996) *Eur J Med Chem* **31**, 439-448.
15 Supuran CT, Scozzafava A, Popescu A, Bobes-Tureac R, Banciu A, Creanga A, Bobes-Tureac G, Banciu MD (1997) *Eur J Med Chem* **32**, 445-452.
16 Alzuet G, Casanova J, Ramirez JA, Borras J, Carugo O (1995) *J Inorg Biochem* **57**, 219-234.
17 Sumalan SL, Casanova J, Alzuet G, Borras J, Castiñeiras A, Supuran CT (1996) *J Inorg Biochem* **62**, 31-39.
18 a) Supuran CT (1995) *Metal Based Drugs* **2**, 327-330; b) Scozzafava A, Supuran CT (1997) *Metal Based Drugs* **4**, 19-26.
19 a) Borras J, Cristea T, Supuran CT (1996), *Main Group Met Chem* **19**, 339-346; b) Jitianu A, Ilies MA, Briganti F, Scozzafava A, Supuran CT (1997) *Metal Based Drugs* **4**, 1-7.
20 a) Supuran CT, Scozzafava A (1997) *J Enzyme Inhibition* **12**, 37-51; b) Supuran CT, Briganti F, Scozzafava A (1997) *J Enzyme Inhibition* **12**, 175-190.
21 Mincione G, Scozzafava A, Supuran CT (1997) *Metal Based Drugs* **4**, 27-34.
22 Alzuet G, Ferrer S, Borras J, Supuran CT (1994) *Roum Chem Quart Rev* **2**, 283-300.
23 Briganti F, Mangani S, Orioli P, Scozzafava A, Vernaglione G, Supuran CT (1997) *Biochemistry* **36**, 10384-10392.
24 Borras J, Casanova J, Cristea T, Gheorghe A, Scozzafava A, Supuran CT, Tudor V (1996) *Metal Based Drugs* **3**, 143-148.
25 Supuran CT, Mincione F, Scozzafava A, Briganti F, Mincione G, Ilies MA (1997) *Eur J Med Chem*, in press.
26 Supuran CT, Scozzafava A, Saramet I, Banciu MD (1997) *J Enzyme Inhibition*, in press.
27 Jitianu A, Ilies MA, Scozzafava A, Supuran CT (1997) *Main Group Met Chem* **20**, 151-156.
28 Forsman C, Behravan G, Osterman A, Jonsson BH (1988) *Acta Chem Scand* **B42**, 314-318.
29 Behravan G, Jonasson P, Jonsson BH, Lindskog S (1991) *Eur J Biochem* **198**, 589-592.
30 Khalifah RG, Strader DJ, Bryant SH, Gibson SM (1977) *Biochemistry* **16**, 2241-2247.
31 Nyman PO, Lindskog S (1964) *Biochim Biophys Acta* **85**, 141-151.
32 Henderson LE, Henriksson D, Nyman PO (1976) *J Biol Chem* **251**, 5457-5463.
33 Maren TH, Wynns GC, Wistrand PJ (1993) *Mol Pharmacol* **44**, 901-906.
34 Young RW, Wood KH, Vaughan JR, Anderson GW (1956) *J Am Chem Soc* **78**, 4649-4654.
35 Pocker Y, Stone JT (1967) *Biochemistry* **6**, 668-678.
36 Baird TT, Waheed A, Okuyama T, Sly WS, Fierke CA (1997) *Biochemistry* **36**, 2669-2678.
37 Maren TH, Bar-Ilan A, Conroy CW, Brechue WF (1990) *Exp Eye Res* **50**, 27-36.
38 Maren TH, Brechue WF, Bar-Ilan A (1992) *Exp Eye Res* **55**, 73-79.
39 Brechue WF, Maren TH (1993) *Invest Ophthalmol Vis Sci* **34**, 2581-2587.
40 Borja P, Alzuet G, Server-Carriò J, Borras J, Supuran CT (1997) *J Biol Inorg Chem (JBIC)*, in press.
41 Supuran CT, Balaban AT, Gheorghiu MD, Schiketanz A, Dinculescu A, Puscas I (1990) *Rev Roum Chim* **35**, 399-405.
42 Supuran CT, Scozzafava A (1997) unpublished results.

**Received: September 24, 1997 - Accepted: October 17, 1997 -**
**Received in revised camera-ready format: October 23, 1997**

